# Suspected rodenticide exposures in humans and domestic animals: Data from inquiries to the Norwegian Poison Information Centre, 2005–2020

**DOI:** 10.1371/journal.pone.0278642

**Published:** 2022-12-08

**Authors:** Arnulf Soleng, Kristin Skarsfjord Edgar, Anita von Krogh, Kristin Opdal Seljetun

**Affiliations:** 1 Department of Pest Control, Norwegian Institute of Public Health, Oslo, Norway; 2 Norwegian Poison Information Centre, Norwegian Institute of Public Health, Oslo, Norway; Sichuan University, CHINA

## Abstract

Rodent control is necessary to prevent damage and spread of disease, and the most common pesticides used for urban and rural rodent control are anticoagulant rodenticides. The aim of this present study was to present data on suspected exposure to rodenticides in humans and domestic animals in Norway based on inquiries to the Norwegian Poison Information Centre in the 16-year period from 2005 through 2020. A total of 4235 inquiries regarding suspected exposures to rodenticides were registered in the study period. Of these, 1486 inquiries involved humans and 2749 animals. Second generation anticoagulants were involved in 68% of human exposures and 79% of animal exposures. Dogs were the most frequent species involved in the animal exposures with 93% of the inquiries, while cats were second most frequent involved. Around 50% of the human inquiries concerned children at the age of 0–4 years. Only 2% of the cases were in the age group 10–19 years, while adults comprised 35% of the inquiries. Acute poisonings accounted for almost 100% of the inquiries among both humans and animals. The exposure was accidental in 99% of the animal exposures and in 85% of the human exposures. In humans, only 14 inquiries were regarding occupational related accidents. Misdeed or self-inflicted injury accounted for 15% of the human inquiries and were the cause of 79% of the severe poisonings. Severe poisoning was only assessed in 1% of the cases involving children under 5 years. In contrast, 17% of the inquiries concerning adults (≥20 years) were assessed as severe. Subsequently, to prevent human and animal rodenticide exposure, we urge the use of non-chemical methods such as sanitation, rodent proofing (a form of construction which will impede or prevent rodents access to or from a given space or building) and mechanical traps. Restricting the use of rodenticides to professional pest controllers (or other persons with authorisation), reinforcing high quality education of these persons, and securing compliance of the best codes of practice could be advocated to reduce accidental exposure to rodenticides in humans and animals.

## Introduction

Different species of rats and mice are ubiquitous and opportunistic rodent pests with worldwide distribution [1]. The major rodent species causing problems in urban areas in Norway is the brown rat (*Rattus norvegicus*). The brown rat also lives in rural areas, typically in connection with sewers, farms, and small villages. Furthermore, in rural areas several species of free-living mice often invade farm-buildings, houses, cabins etc. for shelter during wintertime. This occurs all over the country from sea level to alpine mountain areas depending on species of mice. The invasion of mice typically occurs in early autumn, often beginning in August/September dependent on latitude and altitude. These mice species include wood mouse (*Apodemus sylvaticus*), yellow-necked mouse (*Apodemus flavicollis*), bank vole (*Myodes glareolus*), northern red-backed vole (*Myodes rutilus*), grey red-backed vole (*Myodes rufocanus*), field vole (*Microtus agrestis*) and tundra vole (*Microtus oeconomus*). These species do not reproduce indoors in Norway. However, merely overwintering can cause extensive damage due to gnawing, odour from urine, excrements, and dead animals. The reproduction occurs outside in nature during the spring and summer months. The house mouse (*Mus musculus*), a major rodent pest worldwide, is only found in scattered locations in Norway where it only reproduces indoors. There are only a few unpublished reports of house mice found outdoors in Norway (Reidar Mehl, pers. comm.). In contrast to the free-living species of mice, the house mouse and the brown rat are typically commensal rodent pests living in close contact with humans during their entire life cycle. Another rodent, the European water vole (*Arvicola amphibius*) cause problems in agriculture, gardens, lawns etc and is distributed in most of the country. This species does not enter buildings as the other rodents do. Earlier there were attempts to control this species with anticoagulant rodenticides (ARs), but there is no such use among professional pest controllers in Norway today. The roof rat (*Rattus rattus*) is another major rodent pest species worldwide [1]. The species is extinct in Norway, but has the potential to re-enter, especially from international cargo ship traffic.

The purpose of rodent control is to protect buildings, installations, crops, stored human and animals feed etc. as well as to prevent the spread of rodent vector disease agents. The most common pesticides used for urban and rural rodent control in Norway and worldwide are ARs with different active ingredients [2, 3]. These act by blocking the Vitamin K_1_ cycle, thereby resulting in inability to activate blood-clotting factors. Death occurs from internal haemorrhage [4]. The anticoagulants are classified as first- or second-generation based on the active ingredient. Second generation anticoagulant rodenticides (SGARs) are single dose poisons with a long half-life and higher potency than the first generation (FGARs) products [3]. Mortality from AR poisoning occurs after several days allowing rodents to ingest multiple doses and thereby accumulate high concentrations in their bodies. In addition to ARs, there is still a limited use of the non-anticoagulant alphachloralose by professional pest controllers against mice in Norway. Alphachloralose acts by lowering the metabolism in animals, leading to hypothermia and death if a lethal dose is ingested.

ARs are environmentally persistent and bio-accumulating products, and poisonous to a large range of non-target mammalian wildlife and bird species through both primary and secondary exposure [5–10].

In addition to risk of poisoning and bioaccumulation in wildlife, poisoning with rodenticides is a major concern for domestic animals worldwide [11, 12]. Studies have reported that ARs are the most reported substance causing pet poisonings [13, 14]. A total of 800 calls to the Veterinary Poisons Information Service in Great Britain in 2017 were regarding ARs [15]. Thus, poisoning with rodenticides is a major animal concern. Domestic animals live in close contact with humans and are exposed to similar environmental risk factors. Therefore, it is important to monitor human and animal exposure to rodenticides in a public health and environmental perspective. Exposure to rodenticides in the general human population is primarily through direct contact with the different poison products either by accident or by intention. The incidence of both human and domestic animal poisoning with rodenticides is difficult to assess and is mostly based on national registries.

The aim of this study is to present data on suspected exposures to rodenticides in humans and domestic animals in Norway based on inquiries to the Norwegian Poison Information Centre (NPIC) from 2005 through 2020.

## Materials and methods

The NPIC at the Norwegian Institute of Public Health is an emergency poison control centre that provides advice to the public, kindergartens, veterinarians, hospitals, general practitioners etc. regarding suspected exposures to various toxic agents. The NPIC is open on a 24-hour/7 days basis and is the only poison centre in Norway. The present study is based on inquiries concerning rodenticides from 1^st^ of January 2005 until 31^st^ of December 2020. The following data were recorded for each inquiry: date/time of call, name of product/ingredients, type of exposure (acute/chronic), cause of exposure (accident/self-inflicted/occupational accident), estimated dose or dose range, species (human/animal), and human patient characteristics (sex, age). A risk assessment was performed for all inquiries based on suspected amount of rodenticide (if known) or clinical signs: (1) Poisoning unlikely; (2) Risk of/established mild poisoning; (3) Risk of/established moderate poisoning; (4) Risk of/established severe poisoning; (5) Impossible to assess the danger; (6) Clinical signs not consistent with poisoning.

Following the toxicological risk assessment, advice was given: (1) No treatment or treatment at home; (2) Treatment by general practitioner (GP); (3) Treatment at a hospital; (4) Treatment by veterinarian; (5) Referred elsewhere; (6) General information of toxicity; (7) Other reply.

Note that from May 2017, NPIC reduced the public telephone service regarding animal inquiries (not human inquiries), while maintaining a proper service to veterinarians. Thus, the number of inquiries regarding animals after May 2017 are therefore *per se* not comparable to earlier data.

To test for significant differences in inquiries between years and months we conducted separate Poisson regressions with year and month as variables for humans and animals, respectively. Month was set as categorically variable. This was conducted by the generalized linear model (glm) function in R 4.1.0 [16]. Results were considered significant when P values were 0.05 or lower.

## Results

### Number of inquiries

**Fig 1:**
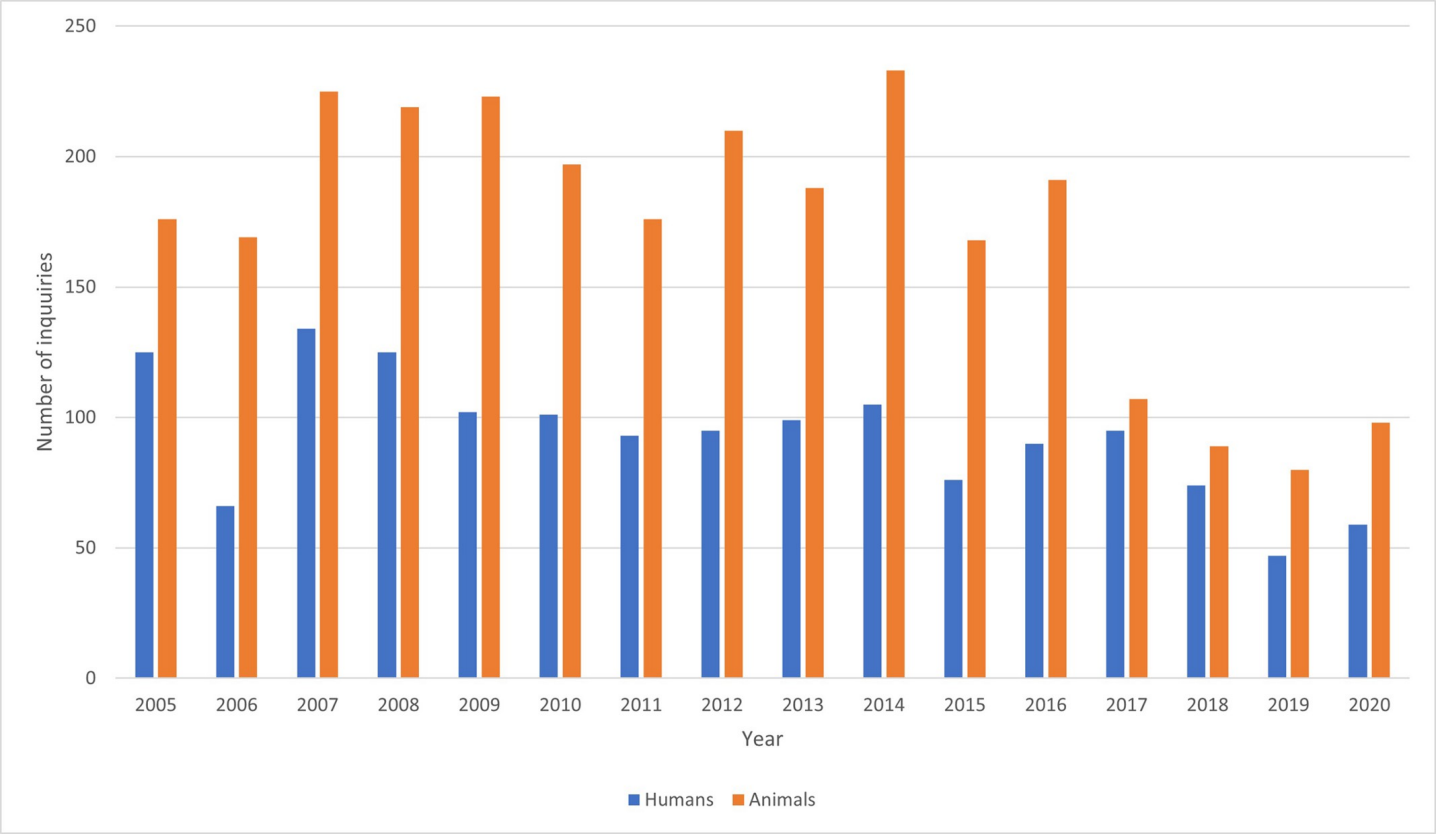
Number of inquiries to the Norwegian Poison Information Centre concerning suspected rodenticide exposures in humans and animals, 2005–2020. Note that from May 2017, NPIC limited the telephone service to a lesser extent responding to inquiries from the public regarding animals.

A total of 4235 inquiries regarding suspected exposures to rodenticides were registered in the 16-year period (Fig 1). This is 0.6% of the total calls to the NPIC in this period (n = 662 494). Of these, 1486 inquiries involved humans and 2749 animals. The mean yearly number of inquiries of rodenticides were 93 (range 47–134) for humans and 172 (range 80–233) for animals. Rodenticides were among the top 5 most frequent inquiries regarding animals to the NPIC and accounted for about 9% of the total inquiries regarding animals in this period (n = 31 587). There were significant differences in number of inquiries between years (P<0.0001) for both humans and animals (Table 1). There was no apparent trend among the human inquiries, but for inquiries concerning animals there was a slight decreasing trend over the years (Fig 1).

**Table 1:** Poisson regression results showing variable estimates, the standard error (Std. Error), Z-value as well as P-value. The regression was run in R version 4.1.0 (R Core Team 2021). Inquiries concerning humans and animals were modelled as dependent variables with month and year as independent variables. Month was set as categorically variable.

Inquiries	Variable	Estimate	Std. Error	Z	P-value
Human	(Intercept)	76.580761	11.421919	6.705	2.02e-11***
	February	-0.057820	0.152121	-0.380	0.703879
	March	0.494970	0.134481	3.681	0.000233***
	April	0.508576	0.134137	3.791	0.000150***
	May	0.567609	0.132685	4.278	1.89e-05***
	June	0.339677	0.138694	2.449	0.014321*
	July	0.670419	0.130319	5.144	2.68e-07***
	August	0.136336	0.145051	0.940	0.347259
	September	0.086075	0.146783	0.586	0.557602
	October	0.488097	0.134657	3.625	0.000289***
	November	0.238751	0.141723	1.685	0.092060
	December	0.174803	0.143770	1.216	0.224043
	Year	-0.037207	0.005677	-6.553	5.62e-11***
Animal	(Intercept)	90.089582	8.424143	10.694	< 2e-16***
	February	-0.188052	0.102513	-1.834	0.066592
	March	0.055570	0.096262	0.577	0.563752
	April	0.055570	0.096262	0.577	0.563752
	May	-0.165324	0.101882	-1.623	0.104652
	June	-0.356675	0.107537	-3.317	0.000911***
	July	0.205852	0.092940	2.215	0.026768*
	August	-0.126752	0.100833	-1.257	0.208737
	September	-0.038840	0.098551	-0.394	0.693501
	October	0.552790	0.086612	6.382	1.74e-10***
	November	0.502217	0.087428	5.744	9.23e-09***
	December	0.129356	0.094583	1.368	0.171424
	Year	-0.043496	0.004188	-10.387	< 2e-16***

Significance codes:

“***” = 0.001,

“**” = 0.01,

“*” = 0.05

### Type of rodenticide

**Fig 2:**
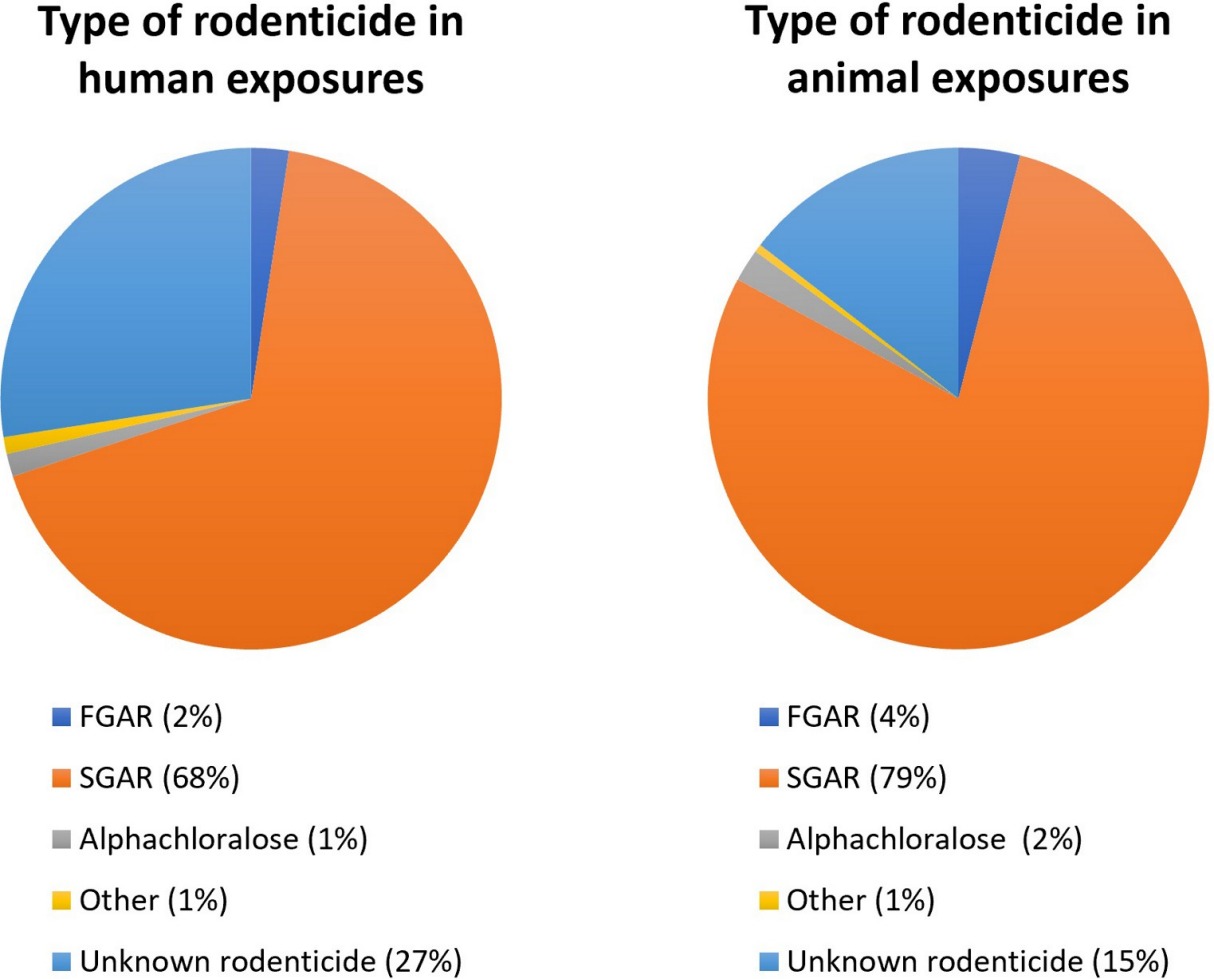
Type of rodenticide involved in human and animal exposures reported to the Norwegian Poison Information Centre, 2005–2020.

Most of the rodenticide exposures were incidents with SGARs with a total of 3173 inquiries. SGARs were reported from 68% of the human exposures and 79% of the animal exposures (Fig 2). In 807 cases (19%), the type of rodenticide was unknown. FGARs were responsible for only 145 inquiries (3%). The non-anticoagulant alphachloralose was the sole rodenticide in a total of 80 inquiries (less than 2%).

A change of inquiries regarding alphachloralose was seen from 2005 to 2020. In the first 11-year period of the study (2005–2015) alphachloralose accounted for only 7 inquiries in total (0.2%). However, in the subsequent 5 years from 2016 to the end of 2020, the total number of inquiries increased to 73 constituting 8% of all rodenticide inquiries in both humans and animals in that period. At the same time, the number of inquiries regarding SGARs decreased from 2634 in 2005–2015 to 539 in 2016–2020. Thus, from 2016 and onwards there has been a decrease in inquiries regarding SGARs, but an increase in inquiries regarding alphachloralose.

### Animal species

Domestic animals were involved in 2749 inquiries. Dogs were the most frequent species involved with 2558 inquiries (93%). Calls regarding cats were second most frequent with 130 inquiries (5%). Horses were represented in 1% of the inquiries, while other animals (livestock, rabbits etc) accounted for 1%.

### Age distribution among humans

**Fig 3:**
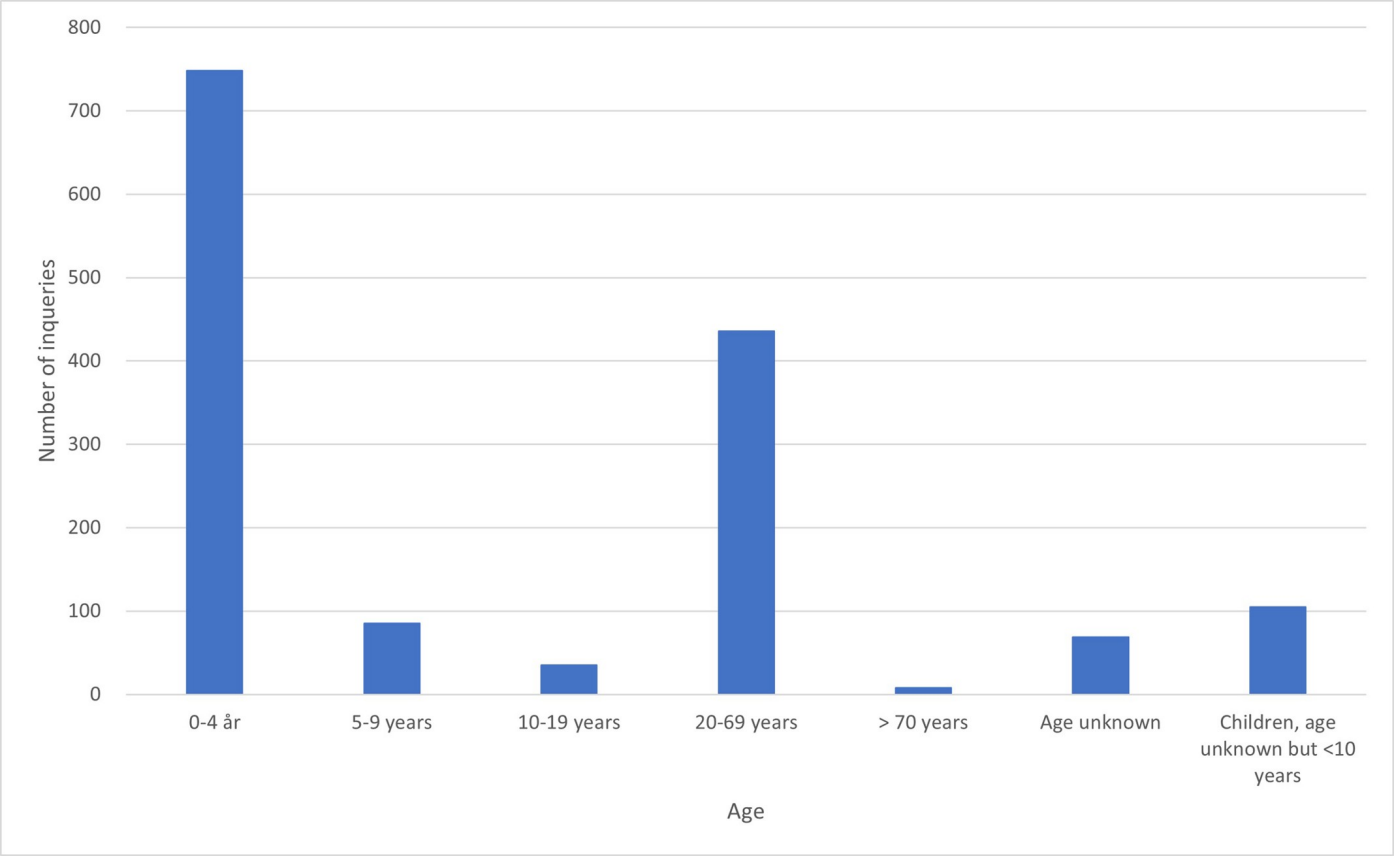
Age distribution in humans concerning suspected rodenticide exposure reported to the Norwegian Poison Information Centre, 2005–2020. Note that “Age unknown” most probably are adults.

Children under the age of 10 years accounted for 938 of 1486 human inquiries (63%). Around 50% of human exposures concerned children at the age of 0–4 years (Fig 3). In the age group 10–19 years, only 35 cases (2%) were reported. Adults (>20 years) comprised of 444 inquiries (30%), while in 69 cases (5%) the age was unknown.

### Risk assessment

**Fig 4:**
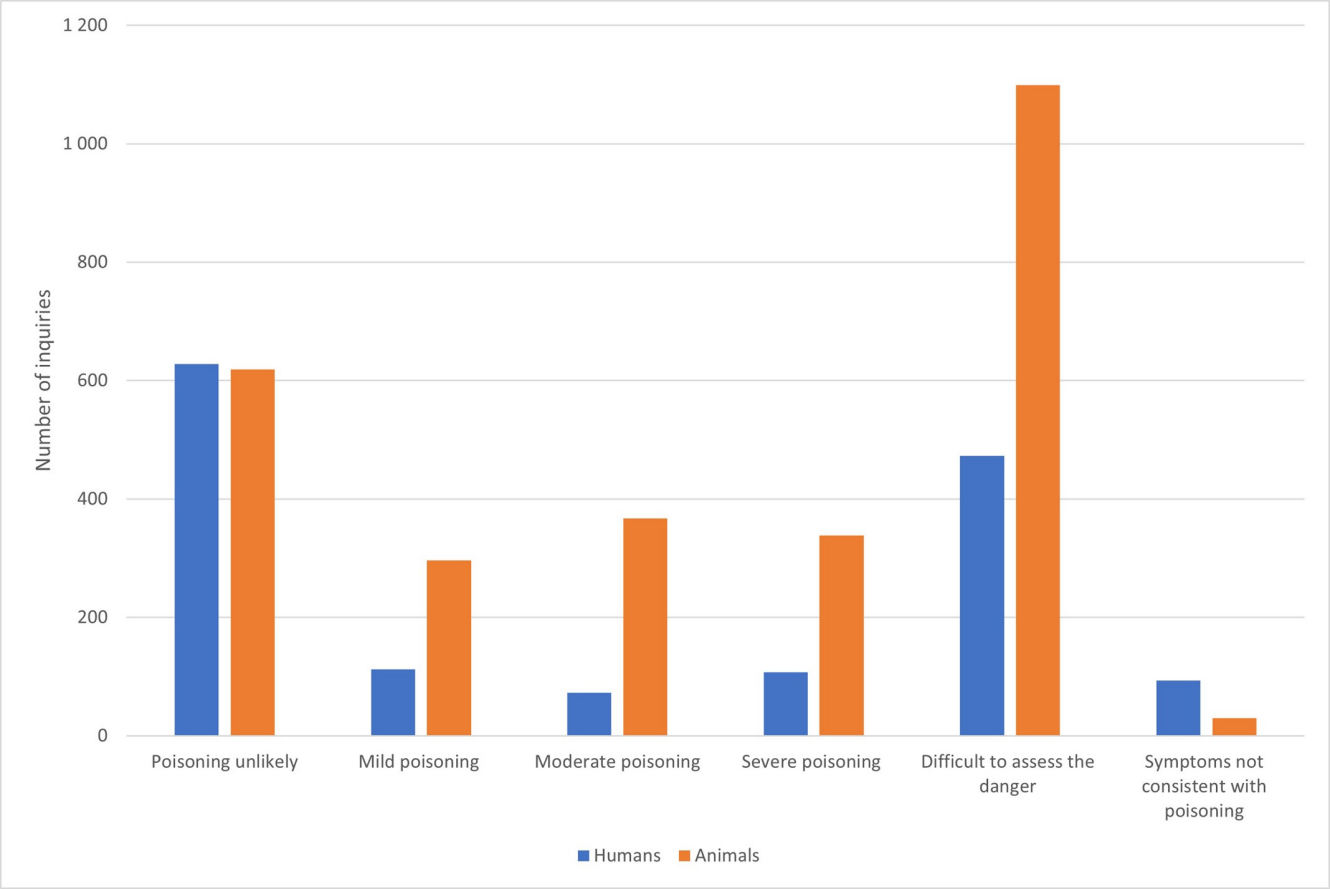
Risk assessment given by the Norwegian Poison Information Centre after inquiries concerning exposure to rodenticides in humans and animals, 2005–2020.

In 628 inquiries regarding humans (42%) and 619 regarding animals (23%) the NPIC considered poisoning to be unlikely (Fig 4). In contrast, 107 inquiries about humans (7%) and 338 about animals (12%) were assessed as risk of severe poisoning. Severe poisoning was only assessed in 1% of the cases involving children under 5 years. In contrast, 17% of the inquiries concerning adults (>20 years) were assessed as severe. It was difficult to assess the risk in 473 human (32%) and 1099 animal (40%) inquiries, respectively. The NPIC registered 11 moderates to severe poisonings due to alphachloralose in animals from 2016 to 2020. At least six of the cases in cats were caused by a suspected secondary poisoning.

### Type and cause of exposure

Acute poisoning accounted for almost 100% of the inquiries. Only 12 human and one animal inquiry were suspected chronic exposures. The exposure was unintentional in 99% of the animal exposures and in 84% of the human exposures. In humans, 14 inquiries were regarding occupational related accidents. Misdeed or self-inflicted injury accounted for 223 human inquiries (15%), and subsequently accounted for 79% of the severe poisonings (Fig 4).

Exposure to rodenticides can occur by ingestion, inhalation, or dermal exposure. Most of the exposures were oral in both humans (84%) and animals (almost 100%). Only 87 inquiries regarding humans were dermal exposures and 66 were inhalation exposures. In 23 inquiries regarding humans the mode of exposure was unknown.

### Recommended treatment advice

**Fig 5:**
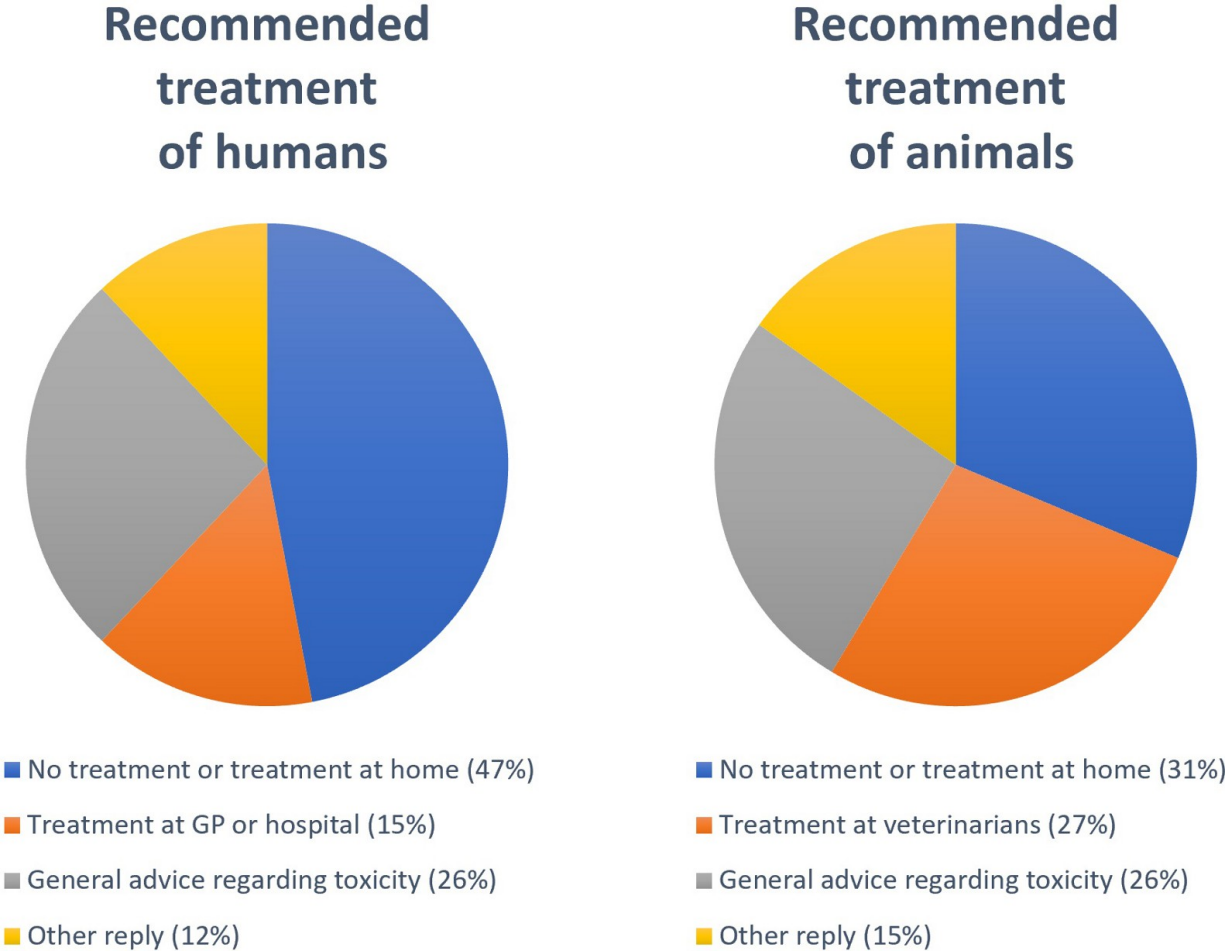
Recommended treatment suggested by the Norwegian Poison Information Centre after inquiries concerning exposure to rodenticides in humans and animals, 2005–2020.

In 47% of the inquiries regarding humans and 31% regarding animals the advice was no treatment necessary or treatment at home (Fig 5). In 217 inquiries (15%) regarding humans the person was advised to seek treatment at GP or hospital, while for animals, 750 cases (27%) were advised to seek veterinary care. General advice regarding toxicity was given in 26% of all inquiries.

### Exposures in relation to month

**Fig 6:**
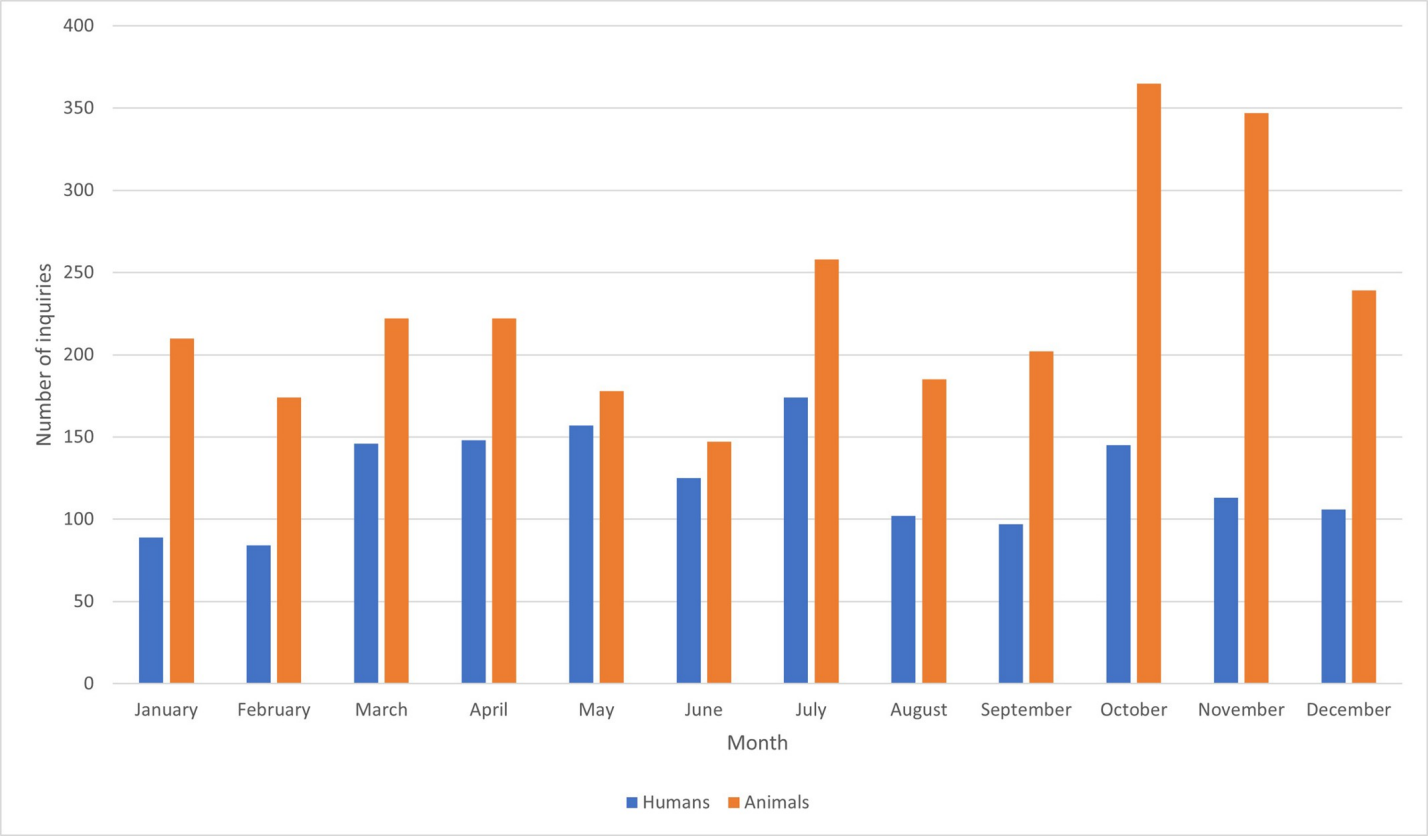
Total number of inquiries per month to the Norwegian Poison Information Centre concerning suspected rodenticide exposures in humans and animals, 2005–2020.

The inquiries were grouped according to month. When comparing the different months to the default month of January, there were significant differences for both human and animal inquiries (Table 1). For human inquiries, March to July as well as October had a significantly higher number of inquiries compared to January (Table 1 and Fig 6). For animal inquiries, July, October and November all had a significantly higher number of inquiries than January, whereas the number of inquiries were significantly lower for June (Table 1 and Fig 6).

## Discussion

Our data demonstrate a high number of suspected exposures to rodenticides in Norway in both humans and dogs, despite strong risk mitigation measures. Most of the inquiries involved acute exposures by direct ingestion of different toxic bait formulations. Baits formulated for rodents are generally cereal based, and made of grains such as oats, wheat, barley, or corn, or a combination thereof. Thus, they are highly palatable and attractive for other species than rodents. Liquid baits and contact dust are not approved formulations in Norway. There is only a limited use of contact foam formulations, placed in rodent tunnels, by professional pest controllers. Colouring agents are used in baits as warning signals to assist in identifying the bait as being toxic. Legislation in Norway demand that all rodenticide baits should contain bittering agents in order to reduce accidental poisoning.

Unintentional ingestions are by far the main reason for inquiries to NPIC regarding rodenticides, constituting 80% of the human inquiries. This is comparable to other studies with 77–96% cases related to accidental ingestion [17, 18]. It is noteworthy that a surprisingly high number of human inquiries (15%) in our study were related to misdeed or self-inflicted injury in adults. Severe outcome is more likely to occur in intentional exposures, where higher doses are ingested. This demonstrates the importance of using tamper resistant, locked, and secured bait stations to avoid both accidents and misuse. The use of cardboard bait boxes, or no bait boxes at all, which both are prohibited, increases the likelihood for both human and animal exposure. Tamper resistant bait boxes makes bait harder to reach, but as rodents hoard food it gives no guaranties of possible relocation of toxic baits. The use of formulations which are difficult to hoard, for example paste and fastened wax blocks instead of grain, pellets, and cellulose bags, might reduce this problem. Loose grain and pellets formulations are only allowed for indoor use by professional pest controllers.

In the present study, children from 0–4 years accounted for approximately half of the human rodenticide inquiries. This is in accordance with a study in France, where this age group constituted 41% of the inquiries about exposure to anticoagulants [17]. In contrast, an American study demonstrated that 88% of the inquiries involved children under the age of 5 years [19]. It is generally believed that small children are more at risk because of their exploratory behaviour, curiosity, and inability to read warning labels. Furthermore, use of different colouring agents as warning signals on rodenticide bait products might have the opposite effect on small children, triggering their curiosity and interest. Strongly coloured toxic bait have been mistaken for being sweets or toys by small children. Interestingly, only 9% of the cases reported by the American Association of Poison Control Centers in 2011–2015 involved adults (>20 years) [19], while they accounted for 30% of the inquiries in our study. Differences between available rodenticide substances and legislation in Europe and USA could contribute to this difference. Luckily, our study demonstrates that only 1% of the exposures in children below 5 years are assessed as severe poisoning. This corresponds to results from poison centres in USA and France [17, 18, 20]. However, data in children is limited by lack of exact measurement of ingested doses. Parents might not have witnessed the ingestion of bait. Thus, the estimation of the dose is often uncertain.

We observed variation from year to year in the number of inquiries. This might reflect changes in the population sizes of small free-living rodents as these are known to fluctuate. Changes is rodent populations between years may lead to differences between years in rodent control efforts. We detected a declining trend in inquiries regarding SGARs in animals from 2015, corresponding to the change in rodenticide legislation in Norway. The main change was restrictions of non-professional use, with a maximum quantity of bait per pack and required use of tamper-resistant bait stations. The public are now restricted to AR products in pre-filled bait stations for indoor use against mice only, and grain and pellet formulations are prohibited for non-professional users. Thus, refilling of bait stations are not allowed for the consumer markets. Certified professional pest controllers have access to a wider variety of rodenticides and are allowed outside use of these products but restricted to use “in close proximity” to buildings or in the sewer. The decline in animal inquiries might also be explained by the fact that NPIC limited the telephone service to a lesser extent responding to inquiries from the public regarding animals. In Denmark, they observed no reduction in AR exposure in stone martens (*Martes foina*) and polecats (*Mustela putorius*) after the regulatory restrictions were implemented [8]. In 2016 (applied in 2018) the European Chemical Agency restricted concentrations of anticoagulants above 0.003% for professional use only, which resulted in even fewer products on the market for the public [21]. Products below this concentration limit can thus be sold to the public. The SGARs are potent enough to be effective below this limit, while the FGARs are not. Subsequently, we get the paradoxical situation that potent bioaccumulating SGAR products can be sold to the public while environmentally friendlier and less toxic FGARs cannot. In our study, FGARs were responsible for only 1% of the inquiries regarding humans and 4% regarding animals.

We detected an overrepresentation of dogs in rodenticide inquiries which is in accordance with previous studies [11, 12, 17, 19]. Dogs have indiscriminate eating habits and are more likely to ingest larger amount of toxic bait. Subsequently we found a higher number of severe poisonings in dogs compared to other species. Cats are picky eaters and are less likely to ingest bait directly, but as efficient predators they are subject to secondary exposure of rodenticides through poisoned prey. Poisoned rodents exhibit abnormal behaviour and slow movements and are thus easy prey for predators [22, 23]. ARs have frequently led to secondary poisoning of non-target animals [24]. Analysis from the Norwegian Veterinary Institute suggest that secondary poisoning of the non-anticoagulant alphachloralose is probable in cats through prey [25]. Despite low number of cases reported to NPIC, our results also demonstrated an increased risk of severe poisoning in cats compared to dogs with this rodenticide. Our study demonstrated an increase in inquiries regarding alphachloralose after 2016 possibly reflecting the expanded restrictions on public use of ARs. Assessment of calls to NPIC involving cats revealed a higher risk of moderate to severe poisoning after exposure to alphachloralose compared to ARs. After several fatal cases in cats, alphachloralose was prohibited for public use in Norway in May 2020, corresponding to the legislation in Sweden [26]. Finland has also made restrictions for private use of alphachloralose, only allowing pre-filled bait stations used indoors against mice [27]. Although rodent control is necessary to protect buildings, crops, stored human and animals feed etc, and to prevent spread of rodent vector disease agents, the risk of poisoning must be balanced against the benefits of rodenticide use. Thus, restrictions in use of FGARs and SGARs due to their bioaccumulating potential in wildlife might result in increased use of other substances with other potential harmful effects.

We found an increased number of inquiries concerning animal exposures during the autumn. This corresponds well with the fact that this is the time-period when free-living mice enter buildings for shelter. Therefore, it is assumed that the use of rodenticides is higher during this season both by professional users as well as public users. Thus, higher use of rodenticides increases the probability of non-target exposure.

Norway has introduced different risk mitigation measures to reduce rodenticide exposures. Governmental education and certification of both professional pest controllers and farmers is considered important. The pest controllers were offered such courses back in 2001, and the official certification was in place in 2004. The system for educating and certifying farmers was in place in 2018. Furthermore, restriction of active ingredients, formulations and mode of use are implemented for different user groups including the public. There is a major focus on sanitation and rodent proofing (a form of construction which will impede or prevent rodents access to or from a given space or building) as the first steps to prevent and reduce rodent problems, use of mechanical traps whenever possible, and injunction of securely fastened tamper resistant bait stations if rodenticides are used. Furthermore, rodenticides used as chemical prevention against future infestation of rodents are prohibited. Thus, rodenticides should only be used when there is an ongoing infestation, and sanitation, rodent proofing, and traps have failed. Despite these risk mitigation measures, there is a need to establish wider study schemes to assess the prevalence and associated risks caused by rodenticides for animals and humans. Law and regulations in both EU and Norway determine the use of rodenticides, and restriction and modality of use are defined to reduce human and animal poisoning. Based on earlier studies demonstrating high prevalence of ARs in wildlife [6, 10], as well as the present study demonstrating exposure among humans and domestic animals, we suggest that the risk mitigation measures to reduce rodenticide exposure have not been effective enough in Norway. Unfortunately, our dataset does not distinguish between professional or public use of rodenticides as the cause for the inquiries to NPIC. However, we detected a reduction of inquiries regarding SGARs in both humans and animals from 2015, corresponding to the increased restriction for public use. Therefore, a total ban of rodenticide use for the public might be considered as a strong and effective risk mitigation measure. Furthermore, certified pest controllers must strive for better compliance with the codes of practice in the industry, and municipalities should perform regular and thorough supervision of these pest controllers and companies.

## Conclusion

In this present study we demonstrate a high number of inquiries regarding rodenticides to the NPIC despite increasing government restrictions in public use of these products. The rodenticide ingestions in humans seldom require medical attention, but as most inquiries involve children, parents experience distress and anxiety by the exposure. We demonstrate a substantial problem with high number of inquiries regarding rodenticides in animals. Subsequently, we urge the use of non-chemical methods such as sanitation, mechanical traps and rodent proofing when controlling rats and mice. Restricting the use of rodenticides to professional pest controllers or other persons with authorisation, reinforcing high quality education in use of rodenticides, and securing compliance of the best codes of practice could be advocated to reduce accidental exposure in humans and animals.

## Supporting information

S1 Data (XLSX)
